# Extragonadal aromatization increases with time after ovariectomy in rats

**DOI:** 10.1186/1477-7827-3-6

**Published:** 2005-01-21

**Authors:** Hong Zhao, Zhanzhuang Tian, Junwei Hao, Boying Chen

**Affiliations:** 1 Department of Neurobiology and Integrative Medicine, Institute of Acupuncture Research (WHO Collaborating Centre for Traditional Medicine, Research Department of Acupuncture), Shanghai Medical College of Fudan University (Formerly Shanghai Medical University), P.O. Box 291, 138 Yi-Xue-Yuan Road, 200032 Shanghai, P. R. China

## Abstract

**Background:**

The circulating estrogen concentration elevated gradually along with time after ovariectomy in rats. To explore the source of the increased circulation estrogen, the extragonadal aromatization as well as the synthesis of androgen in the adrenal cortex of the ovariectomized rats was evaluated.

**Methods:**

Female rats were divided into twelve groups: 1 month after ovariectomy (OVX1M), OVX2M, OVX3M, OVX4M, OVX5M, OVX6M; intact 1 month (INT1M), INT2M, INT3M, INT4M, INT5M, INT6M. The blood concentration of testosterone (T) was measured by radioimmunoassay. The mRNA expressions of P450 aromatase in the liver and subcutaneous abdominal (SA) adipose as well as the adrenal cytochrome P450 17 alpha hydroxylase/lyase (P450c17) were semiquantified by RT-PCR. The P450 aromatase protein expressions in the liver and SA adipose were detected by Western blot.

**Results:**

The blood E2 concentrations increased gradually along with time after ovariectomy in the rats. The 58-kDa aromatase protein and mRNA expressions normalized to β-actin in the OVX6M rats' SA adipose tissues showed higher levels than those from corresponding tissues in the INT6M (p < 0.05). And the ratios of aromatase mRNA and protein to β-actin in the OVX6M rats' liver tissues increased significantly compared with those in the OVX1M rats (p < 0.05). The ratio of adrenal P450c17 to beta-actin in the OVX6M increased markedly, and was higher than OVX1M (p < 0.05), though the blood concentration of T decreased significantly in all the ovariectomized rats (p < 0.05).

**Conclusion:**

Both the subcutaneous abdominal adipose tissues and the liver tissues contributed to the extragonadal aromatisation to promote the circulating E2 levels in the rats along with time after ovariectomy; the adrenal compensation might also be activated naturally.

## Background

Ovaries are the primary source of estrogen. In ovariectomized rats, the production of estrogen is shifted from ovary to a number of extragonadal sites [1,2]. Simpson developed the intriguing concept of extragonadal aromatization, i.e. androgens, particularly androstenedione produced primarily in the adrenal glands, can be converted (aromatized) into estrogens at extraglandular sites, including the mesenchymal cells in adipose tissue and skin, osteoblasts and chondrocytes in bone, vascular endothelial, aortic smooth muscle cells as well as numerous sites in brain [2,3]. Estrogen synthesised within these sites is biologically active in a paracrine or intracrine fashion, although it may escape the local metabolism and enter the circulation [2,3].

Estrogen production in extragonadal sites is dependent on an external source of C19 androgenic precursors [4]. Circulating levels of testosterone (T) and androstenedione as well as dehydroepiandrosterone (DHEA) and DHEA sulphate (DHEAS) become extremely important in terms of providing adequate substrate for estrogen biosynthesis in extragonadal sites. It is now recognized that much of the physiology of androgens is consistent with the concept that T functions as a circulating pro-hormone. In the postmenopausal woman, circulating T levels are an order of magnitude greater than circulating estrogen levels, which by itself suggests the importance of T for maintaining local estrogen levels in extragonadal sites [2]. 65–75% of the circulating T is formed peripherally from androstenedione, DHEA and DHEAS secreted by the adrenals, which form a large precursor reservoir that is available for conversion to T and finally to estrogen [2]. Thus in the species of rat, it is still unknown that whether it is the same situation of T in extragonadal aromatization.

We have observed that the release of corticotrophin-releasing hormone (CRH) in the hypothalamic paraventricular nucleus of the ovariectomized rats increased significantly compared with the intact rats [5]. And more interestingly, the hypothalamic CRH expressions showed a gradual elevation along with time after ovariectomy (submitted data), which suggested that the activity of hypothalamus-pituitary-adrenal axis (HPAA) might promote. These observations lead us to hypothesize that more androgens from adrenal cortex might gradually be converted into estrogens by extragonadal aromatase in the rats along with time after ovariectomy. To test the hypothesis, we measured the blood concentrations of estradiol (E_2_) and T as well as the aromatase expressions in adipose and liver tissues in the ovariectomized rats.

## Methods

### Animals

Female Sprague-Dawley rats (180–200 g), with regular 4-day estrus cycles were purchased from Medical Experimental Animals Centre of Fudan University (Shanghai, China). The animals were housed under laminar flow in an isolated room with controlled temperature and at a 12 /12 (light /dark) schedule. Forty-eight of them underwent ovariectomy with ether anaesthesia, which were then divided randomly into six groups: 1 month after ovariectomy (OVX1M), OVX2M, OVX3M, OVX4M, OVX5M, OVX6M. The corresponding control groups were: intact 1 month (INT1M), INT2M, INT3M, INT4M, INT5M, INT6M. All experimental procedures involving the use of animals were conducted in accordance with NIH Guidelines and were reviewed and approved by the Animal Use and Care Committee for the Fudan University.

### Tissue collection and preparation

At the time of sacrifice, the vaginal cytology of each rat was first examined. The tissues of the rats were collected respectively, and those of the intact control animals, during the period of diestrous. All the operations were carried out at 4°C. The liver tissues, subcutaneous abdominal (SA) adipose tissues and the adrenals were excised, and then snap-frozen in liquid nitrogen, and stored at -80°C. The preparation of the microsomal pellet was accordance with the report by Hiroshi [6]. Total tissue RNA was extracted using 'TRIzol Regent' (Biobasic Inc, Canada), and the purity and integrity of the RNA were checked spectroscopically and by gel electrophoresis before analytical procedures.

### Semiquantitative RNA analysis

To compare the level of adrenal cytochrome P450 17α hydroxylase/lyase (P450c17), liver and SA adipose P450arom expressions after different treatments, PCR methologies were adapted to provide a semiquantitative measure of mRNA levels. Primers were synthesized based on published reports [7,8]. Table 1 summarizes the anticipated size of PCR products, sense and antisense sequences, and locations of primers. Total RNA (2 μg) was transcribed in reverse, in a final volume of 20 μl, using 200IU M-MLV reverse transcriptase in the presence of 25 pmol downstream primer (Sangon Inc), 0.5 mM deoxy-NTP and 20IU Rnasin (from Promega) for 60 min at 42°C before heat denatured for 5 min at 95°C. The cDNAs obtained were further amplified by PCR using 25 pmol of upstream primer (Sangon Inc). We first determined the linear range of amplification of cDNA using each of the primer sets, and then chose an appropriate amplification cycle within this range for each cDNA species. For P450arom and P450c17, we used 35 PCR amplification cycles, and 20 cycles for β-actin gene expression. Each PCR reaction underwent an amplification regimen characterized by (P450arom: 1 min at 94°C, 1 min at 60°C, 2 min at 72°C; P450c17: 1.5 min at 72°C, 2 min at 56°C, 4 min at 72°C) with Taq DNA polymerase (3U per tube) and 2.2 mM magnesium chloride (from Promega) in a final volume of 50 μl. To check the presence of DNA contamination, RT-PCR was performed on 2 μg of total RNA without M-MLV reverse transcriptase (negative control). An internal control (water instead of RNA) for each RT-PCR was performed to investigate RNA contamination of the mixture. For each sample 5 μl of the PCR amplification products were analysed on 2% agarose gels and stained with ethidium bromide. The intensities of the bands were evaluated using the Image Master Software (SYDR-1990, SYNGENE, U.S.A.). The RT-PCR products were extracted and purified from agarose gel by Golden Beads Gel Extraction kit (Sangon Inc., China) and sequenced using radioactive dideoxychain terminating method (Sangon Inc., China).

**Table 1:** Properties of oligonucleotide primers used for PCR.

Target mRNA	Size of PCR products (bp)	Sense (S) or antisense (AS)	Primer sequence (location)
P450c17	299	S AS	5'-TCATCAAGAAGGGAAAAGAA-3' 5'-TGAAGCAGATAGCACAGATG-3'
P450arom	289	S AS	5'-GCTTCTCATCGCAGAGTATCCGG-3' 5'-CAAGGGTAAATTCATTGGGCTTGG-3'
β-actin	550	S AS	5'-AAGCAGGAGTATGACGAGTCCG-3' 5'-GCCTTCATACATCTCAAG TTGG-3'

### Western blotting

A 50 μg sample of the microsomal protein was loaded into each lane along with a prestained protein size marker (Bio-Rad Laboratories, Inc., Hercules, CA), electrophoresed on a 10% SDS-polyacrylamide gel at 18 V/cm, and electroblotted onto a polyvinylidene difluoride membrane (Micron Separations, Westboro, MA) using a wet electroblotter. After blocking in fat-free milk, incubation was conducted with the antiaromatase antibody (1:200; Boster Biological Technology LTD., China) and β-actin antibody (1:3000) at room temperature in 18°C for 4 h in TBS-T solution (20 mM Tris, 137 mM NaCl, and 0.1% Tween-20, pH 7.6). After extensive washing, blots were incubated with AP-labelled goat antirabbit antiserum (Sino-American Biotechnology Co., China) for 60 min at room temperature in 18°C and developed using NBT/BCIP detection system (Amersham Pharmacia Biotech). The intensities of the bands were evaluated using the Image Master Software (SYDR-1990, SYNGENE, U.S.A.), and values were normalized to β-actin immunoreactivity in each sample and expressed as percent of the control. Specificity of the aromatase immuno-staining was determined by preincubation of antiserum for 24 h at 4°C with varying concentrations of aromatase, with the primary antibody omitted to identify non-specific staining as well.

### RIA of blood estrogen and testosterone concentrations

At the time of sacrifice, the blood samples (0.8 ml) of the rats were collected from tail veins respectively, and the corresponding intact controlling animals, during the period of diestrous. The plasma was separated by centrifugation and stored at -70°C until assayed. Concentration of blood hormones were determined by double-antibody RIA kits purchased from the National Atomic Energy Research Institute (Beijing, China.). The samples were assayed in duplicate, and all the subjects' samples were assayed together. The sensitivity of the kit was 0.8 pg/ml (testosterone) and 1.4 pg/ml (estrogen), the intra- and interassay coefficients of variation, 3.7–8.0% and 4.74–7.7%.

### Statistical analysis

All data are presented as means ± S.E.M. Statistical analysis was performed on raw data using two-way analysis of variance (ANOVA), with the significance set at p < 0.05 and p < 0.01 in two-tailed testing chosen.

## Results

### Vaginal cytology of the animals

The epithelial cells were stained by haematoxylin-eosin (HE). The intact rats (INT1M, 2M, 3M, 4M, 5M, 6M) showed regular 4-day estrus cycle change. The cyclic change disappeared in the ovariectomized (OVX1M, 2M, 3M, 4M, 5M, 6M) rats. A few of mature vaginal epithelia were observed in the smears of the OVX5M and OVX6M rats, and the percent of mature epithelia increased significantly in the OVX6M rats (p < 0.05) (Table 2).

**Table 2:** The percent of mature vaginal epithelia and the blood concentrations of E_2 _and T of the rats

Groups		The percent of mature vaginal epithelia	Blood E_2 _level (pg/ml)	Blood T level (pg/ml)
1M	INT	(15.6 ± 0.88)%	56.60 ± 14.13	27.92 ± 1.74
	OVX	(0.19 ± 0.019)%*	4.06 ± 1.36*	18.91 ± 2.53*
2M	INT	(15.8 ± 0.91)%	59.12 ± 11.23	26.39 ± 2.04
	OVX	(0.17 ± 0.015)%*	8.94 ± 1.78*	18.03 ± 2.20*
3M	INT	(14.8 ± 0.90)%	58.25 ± 13.93	28.86 ± 2.19
	OVX	(0.20 ± 0.025)%*	16.72 ± 3.71*	20.21 ± 1.96*
4M	INT	(15.1 ± 0.86)%	59.35 ± 10.35	26.99 ± 2.14
	OVX	(0.19 ± 0.020)%*	28.10 ± 8.88*^#^	20.89 ± 2.69*
5M	INT	(16.4 ± 1.01)%	63.34 ± 15.85	27.03 ± 1.93
	OVX	(0.26 ± 0.034)%* ^#^	28.97 ± 6.61*^#^	21.35 ± 2.33*
6M	INT	(16.8 ± 1.06)%	59.15 ± 13.34	29.36 ± 2.56
	OVX	(0.29 ± 0.027)%* ^#^	31.05 ± 6.61*^#^	21.54 ± 2.09*

*p < 0.05 vs corresponding intact; # p < 0.05 vs OVX1M

### Blood concentrations of estrogen and testosterone

The blood E_2 _concentrations decreased significantly in the OVX1M, 2M and 3M (p < 0.01) compared with those in the INT1M, 2M and 3M. The concentrations in OVX4M, 5M and 6M groups increased significantly (p < 0.05) compared with OVX1M, though still lower than INT4M, 5M and 6M. There were no disparities between the INT1M, 2M, 3M, 4M, 5M and 6M groups (Table 2). The blood T concentrations decreased significantly in the OVX groups compared with corresponding intact controls (p < 0.05). Though there were slightly increases in the OVX3M, 4M, 5M and 6M compared with OVX1M, no statistical significances were detected (Table 2).

### RT-PCR analysis: Effects of ovariectomy on adrenal P450c17, liver and SA adipose P450arom mRNA levels

**Figure 1:**
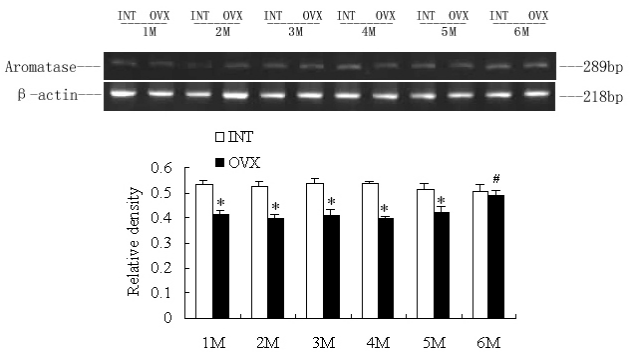
**RT-PCR analysis of liver P450arom mRNA expressions of the rats. **The upper picture shows the gel electrophoresis of the RT-PCR products for the liver P450arom. Densitometric analysis of the mRNA concentration using PCR product/β-actin expressed as the mean with SEM bar in each column indicated in the lower panel. * p < 0.05 vs corresponding intact controls, # p < 0.05 vs OVX1M.

**Figure 2:**
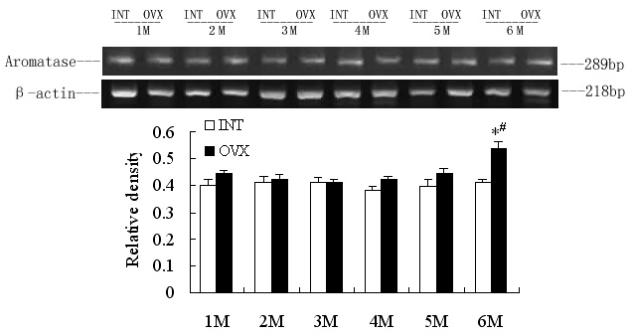
**RT-PCR analysis of SA adipose P450arom mRNA expressions of the rats. **The upper picture shows the gel electrophoresis of the RT-PCR products for the SA adipose P450arom. Densitometric analysis of the mRNA concentration using PCR product/β-actin expressed as the mean with SEM bar in each column indicated in the lower panel. * p < 0.05 vs corresponding intact controls, # p < 0.05 vs OVX1M.

**Figure 3:**
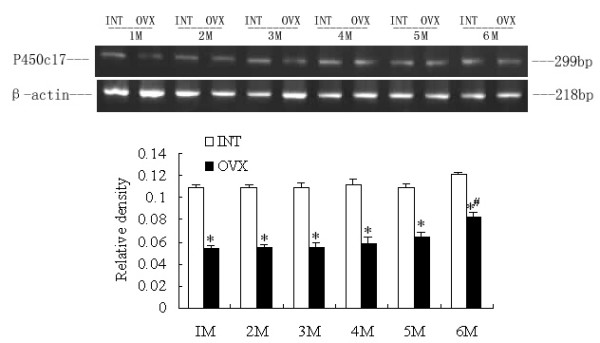
**RT-PCR analysis of adrenal P450c17 mRNA expressions of the rats. **The upper picture shows the gel electrophoresis of the RT-PCR products for the adrenal P450c17. Densitometric analysis of the mRNA concentration using PCR product/β-actin expressed as the mean with SEM bar in each column indicated in the lower panel. * p < 0.05 vs corresponding intact controls, # p < 0.05 vs OVX1M.

Comparison of the amplified PCR fragments with rat P450c17 and ovary aromatase cDNA sequences revealed 100% homology (data not shown). Densitometric analysis of the mRNA concentration using target product/β-actin was expressed as the mean with SEM. The ratios of liver P450arom to β-actin in the OVX1M, 2M, 3M, 4M and 5M groups were lower than the corresponding intact controls (p < 0.05). The ratio increased significantly in the OVX6M compared with OVX1M (p < 0.05), and no difference was detected between OVX6M and INT6M (Fig 1). The ratios of SA adipose P450arom to β-actin in the OVX1M, 2M, 3M, 4M and 5M groups appeared no significant changes compared with those in the corresponding intact control, and the ratio in the OVX6M was higher than that in all other groups (p < 0.05) (Fig 2). The ratio of adrenal P450c17 to β-actin in the OVX1M decreased significantly compared with that in the INT1M (p < 0.05), and those in the OVX4M and OVX5M increased slightly, with statistical disparities between OVX4M and INT4M, or OVX5M and INT5M. In the OVX6M, the ratio increased markedly, and was higher than OVX1M (p < 0.05) (Fig 3). However, no changes occurred on the ratios of adrenal P450c, liver and SA P450arom among INT1M, 2M, 3M, 4M, 5M and 6M(Fig 1, 2,3).

### Western blot analysis: Effects of ovariectomy on liver and SA adipose P450arom protein levels

**Figure 4:**
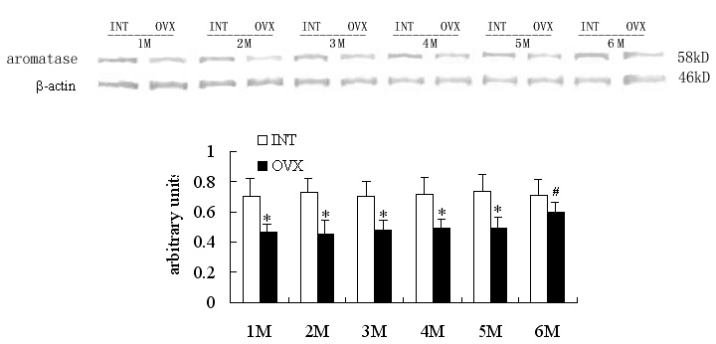
**Western blot analysis of liver P450arom expressions of the rats. **The upper picture shows the Western blot analysis of the liver aromatase P450. Densitometric analysis of the protein concentration using aromatase/β-actin expressed as the mean with SEM bar in each column indicated in the lower panel. * p < 0.05 vs corresponding intact controls, # p < 0.05 vs OVX1M.

**Figure 5:**
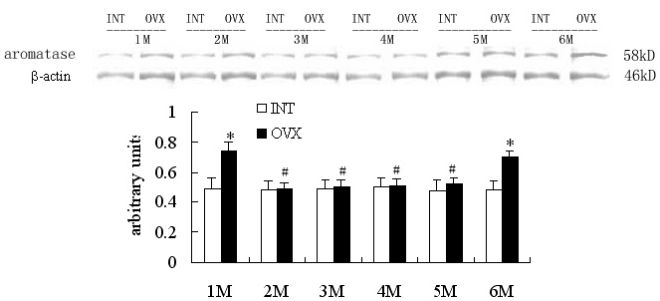
**Western blot analysis of SA adipose P450arom expressions of the rats. **The upper picture shows the Western blot analysis of the SA adipose aromatase P450. Densitometric analysis of the protein concentration using aromatase/β-actin expressed as the mean with SEM bar in each column indicated in the lower panel. * p < 0.05 vs corresponding intact controls, # p < 0.05 vs OVX1M.

Densitometric analysis of the protein concentration using aromatase/β-actin was expressed as the mean with SEM. The ratios of liver P450arom to β-actin in the OVX groups were lower than the corresponding intact controls (p < 0.05). But the ratio increased significantly in the OVX6M compared with OVX1M (p < 0.05) (Fig 4). The ratios of SA adipose P450arom to β-actin in the OVX1M and OVX6M increased significantly compared with those in the INT1M and INT6M (p < 0.05). And the ratios in the OVX2M, 3M, 4M and 5M produced no disparities compared with corresponding intact controls (Fig 5). No disparities were detected among INT1M, 2M, 3M, 4M, 5M and 6M (Fig 4, 5). No immunoreactive bands detected in the samples when using antiserum after preabsorption with excessive antigens and omission of the primary antibody.

## Discussion

The most interesting findings in the present study were that the circulating E_2 _levels increased gradually along with time after ovariectomy in the rats. If ovary is not the source of estrogen in ovariectomized rats, then the question arises as to the origin of the estrogens found in the circulation. Extragonadal aromatization has been in a general sense recognized, although its significance is only becoming to be appreciated, as will be explained further.

It has been reported that aromatization in the adipose tissue is not negligible under normal and pathological conditions [9]. Due to the highest conversion of C19 precursor such as androstenedione to estrogen was observed in adipose tissue obtained from the subcutaneous abdominal wall but not from intraperitoneal cavity [10], our research focused on the aromatase activity of the SA adipose tissues. In the present results, increased aromatase protein and mRNA expressions were observed in the OVX6M rats' SA adipose tissue. Hemsell and co-workers [11] first addressed the significance of adipose tissue as a major source of estrogen production, i.e. there is a progressive increase in the conversion efficiency with advancing age, and the increase of estrogen production as a function of obesity [12-14]. The body fat of ovariectomized rats increases significantly [15], and the mesenchymal cells from the SA adipose tissue could be active, which might be closely related to the higher aromatase expression in the OVX6M. Yet, only in the OVX6M was there an associated significant increase of the aromatase expression in the SA adipose tissue, but not in other OVX groups, which by itself suggested that the elevated expression of aromatase in the OVX6M was not closely implicated in the obesity of the rats. We have also observed that the content of hypothalamic CRH increased along with time after ovariectomy in the rats, with a significant elevation in the OVX6M (submitted data). It has been reported that CRH may regulate the expressions of extragonadal aromatase via CRH receptor-II [16], which may help us understand the involved mechanism of the enhanced SA adipose aromatization and the eventual increased E_2 _in the OVX6M rats, but further studies are earnestly needed. However, in the present results, it's confusing that the protein but not mRNA expression of SA adipose aromatase in the OVX1M showed an elevation compared with that in the INT1M. This might provide evidence indicating the inconsistency on the levels between transcription and translation of the aromatase gene.

The factors possibly involved in the regulation of aromatase expression are still poorly understood [17]. Many studies have been performed to assess the possible dependence of the enzyme on androgens, though the data available are conflicting [17,18]. The situation is complicated by the fact that, in postmenopausal women, only about 25% of circulating T is derived by direct secretion from the ovaries [2]. The rest is formed largely from circulating precursors derived from the adrenal cortex. In the present study, the blood concentrations of T in the rats were detected. In the OVX groups there were decreases of the T level compared with corresponding intact ones, and no significant changes occurred along with the time after ovariectomy. These might be implicated in the less importance of T in the extragonadal aromatisation in rats than in women. Nevertheless, it's the first time to present the dynamic changes of T levels in the rats after ovariectomy. But further explorations are still needed on the way by which the aromatase enzyme expression is activated in ovariectomized rats.

The microsomal enzyme cytochrome P450c17 is an important regulator of steroidogenesis. The enzyme has two functions: 17alpha-hydroxylase and 17,20-lyase activities. These functions determine the ability of adrenal glands and gonads to synthesize sex steroids (17,20-lyase activity) [19]. Its activity is abundant in testis, and lesser in ovary, and low levels of P450c17 activity in adrenals [20]. It is well known that adrenal is the principle organ to secrete sexual hormones except ovarian in females [21]. In the present study, due to the T levels did not show significant change along with the time after ovariectomy, the mRNA expressions of P450c17 were semi-quantified by RT-PCR to indirectly report the androgen synthesis in the adrenals. Excitedly, the ratio of adrenal P450c17 to β-actin in the OVX4M and OVX5M increased slightly, and in the OVX6M, the ratio increased markedly, which was higher than OVX1M. These suggested that the androgen synthesis activity of adrenal might be enhanced, and the more androgens might secrete from adrenal cortex.

Though it has been reported that the splanchnic tissue is a minor site for extragonadal aromatization of androgens [22], there is a significant conversion of androstenedione to estrone by liver tissues [15]. In adult liver homogenates, C19 norsteroid (19-nortestosterone; NT) is readily aromatized to estrogens [23,24]. The present results showed that the aromatase expressions (mRNA and protein) of the liver tissue in the OVX1M, 2M, 3M, 4M and 5M groups were lower than the corresponding intact controls, and in the OVX6M the ratio increased significantly compared with that in the OVX1M. On one hand, after ovariectomy, the diminution of C19 precursor from ovaries for aromatization may induce the decreased expressions of aromatase in the OVX rats compared with the gonad-intact rats in our results. On the other hand, the results suggested that aromatization of liver tissue enhanced six months after ovariectomy. However, in the present study, the aromatase expressions in the OVX rats did not show consistent changes between the SA adipose and liver tissues, which may suggest that the role of SA adipose and splanchnic tissues in the extraglandular aromatisation might be different. Nonetheless, in the final analysis, our results suggested that both the SA adipose and liver tissues contributed to the extragonadal aromatisation to promote the circulation estrogen concentrations.

## Conclusions

Both the subcutaneous abdominal adipose tissues and the liver tissues contributed to the extragonadal aromatization to promote the circulating E_2 _levels in the rats along with time after ovariectomy; the adrenal compensation might also be activated naturally.

## Authors' contributions

Hong Zhao and Zhanzhuang Tian designed the study, performed the studies and the statistical analysis, and drafted the manuscript. Junwei Hao performed the animal experiment. Boying Chen conceived of the study, and participated in its design and coordination. All authors read and approved the final manuscript.

## References

[B1] Simpson ER, Susan RD (2001). Minireview: Aromatase and the Regulation of Estrogen Biosynthesis – Some New Perspectives. Endocrinology.

[B2] Simpson ER (2003). Sources of estrogen and their importance. J Steroid Biochem Mol Biol.

[B3] Simpson ER (2000). Role of aromatase in sex steroid action. J Mol Endocrin.

[B4] Samuel SC, Robert BJ, Robert LB (2001). Reproductive Endocrinology.

[B5] Zhao H, Tian ZZ, Chen BY (2003). Increased corticortropin-releasing hormone release in ovariectomized rats' paraventricular nucleus: effects of electroacupuncture. Neurosci Lett.

[B6] Sumitani H, Shozu M, Segawa T, Murakami K, Yang HJ, Shimada K, Inoue M (2000). In situ estrogen synthesized by aromatase p450 in uterine leiomyoma cells promotes cell growth probably via an autocrine/intracrine mechanism. Endocrinology.

[B7] Bagna B, Narender K, Russell MK, Allen HG, Kalyan S (2000). Estrogen Receptor-β Expression in Relation to the Expression of Luteinizing Hormone Receptor and Cytochrome P450 Enzymes in Rat Ovarian Follicles. Biol Reprod.

[B8] Levallet J, Bilinska B, Mittre H, Genissel C, Fresnel J, Carreau S (1998). Expression and immunolocalization of functional cytochrome P450 aromatase in mature rat testicular cells. Biol Reprod.

[B9] Vague J, Sardo (1981). Aromatization of androgens (author's transl). Sem Hop.

[B10] Simpson ER, Mahendroo MS, Means GD, Kilgore MW, Hinshelwood MM, Graham_Lorence S, Amarneh B, Ito Y, Fisher CR, Michael MD (1994). Aromatase cytochrome P450, the enzyme responsible for estrogen biosynthesis. Endocrin Rev.

[B11] MacDonald PC, Edman CD, Hemsell DL, Porter JC, Siiteri PK (1978). Effect of obesity on conversion of plasma androstenedione to estrone in postmenopausal women with and without endometrial cancer. Am J Obst Gynecol.

[B12] Bray GA (2002). The underlying basis for obesity: relationship to cancer. J Nutr.

[B13] Frost PG, Reed MJ, James VH (1980). The aromatization of androstenedione by human adipose and liver tissue. J Steroid Biochem.

[B14] Grodin JM, Siiteri PK, MacDonald PC (1973). Source of estrogen production in post-menopausal women. J Clin Endocrin Metab.

[B15] Davidge ST, Zhang YL, Stewart KG (2001). A comparison of ovariectomy models for estrogen stidies. Am J Physiol Regul Integ Cop Physiol.

[B16] Calogero AE, Burrello N, Negri CP (1996). Effects of corticotropin-releasing hormone on ovarian estrogen production in vitro. Endocrinology.

[B17] Negri-Cesi P, Colciago A, Motta M, Martini L, Celotti F (2001). Aromatase expression and activity in male and female cultured rat hypothalamic neurons: effect of androgens. Mol Cel Endocrin.

[B18] Beyer C, Hutchison JB (1997). Androgens stimulate the morphological maturation of embryonic hypothalamic aromatase-immunoreactive neurons in the mouse. Brain Res Dev Brain Res.

[B19] Fevold HR, Lorence MC, McCarthy JL, Trant JM, Kagimoto M, Waterman MR, Mason JI (1989). Rat P45017a from testis: characterization of a full-length cDNA encoding a unique steroid hydroxylase capable of catalyzing both the D4- and D5-steroid 17,20-lyase reactions. Mol Endocrin.

[B20] Schlinger BA, Lane NI, Grisham W, Thompson L (1999). Androgen synthesis in a songbird: a study of cyp17 (17alpha-hydroxylase/C17, 20-lyase) activity in the zebra finch. Gen Comp EndocrinJan.

[B21] Meikle AW, Daynes RA, Araneo BA (1991). Adrenal androgen secretion and biologic effects. Nor Am Endocrin Metab Clin.

[B22] Longcope C, Billiar RB, Takaoka Y, Reddy PS, Richardson D, Little B (1983). Tissue sites of aromatization in the female rhesus monkey. Endocrinology.

[B23] Harada N, Ota H, Yoshimura N, Katsuyama T, Takagi Y (1998). Localized aberrant expression of cytochrome P450 aromatase in primary and metastatic malignant tumors of human liver. J Clin Endocrin Metab.

[B24] Yoshiji S, Yamamoto T, Okada H (1986). Aromatization of androstenedione and 19-nortestosterone in human placenta, liver and adipose tissues. Nippon Naibunpi Gakkai Zasshi.

